# Spatial Accessibility to Health Care Services: Identifying under-Serviced Neighbourhoods in Canadian Urban Areas

**DOI:** 10.1371/journal.pone.0168208

**Published:** 2016-12-20

**Authors:** Tayyab Ikram Shah, Scott Bell, Kathi Wilson

**Affiliations:** 1 School of Physical Therapy, College of Medicine, University of Saskatchewan, Saskatoon, Saskatchewan, Canada; 2 Department of Geography and Planning, University of Saskatchewan, Saskatoon, Saskatchewan, Canada; 3 Department of Geography, University of Toronto Mississauga, Mississauga, Ontario, Canada; Fundacao Oswaldo Cruz, BRAZIL

## Abstract

**Background:**

Urban environments can influence many aspects of health and well-being and access to health care is one of them. Access to primary health care (PHC) in urban settings is a pressing research and policy issue in Canada. Most research on access to healthcare is focused on national and provincial levels in Canada; there is a need to advance current understanding to local scales such as neighbourhoods.

**Methods:**

This study examines spatial accessibility to family physicians using the Three-Step Floating Catchment Area (3SFCA) method to identify neighbourhoods with poor geographical access to PHC services and their spatial patterning across 14 Canadian urban settings. An index of spatial access to PHC services, representing an accessibility score (physicians-per-1000 population), was calculated for neighborhoods using a 3km road network distance. Information about primary health care providers (this definition does not include mobile services such as health buses or nurse practitioners or less distributed services such as emergency rooms) used in this research was gathered from publicly available and routinely updated sources (i.e. provincial colleges of physicians and surgeons). An integrated geocoding approach was used to establish PHC locations.

**Results:**

The results found that the three methods, Simple Ratio, Neighbourhood Simple Ratio, and 3SFCA that produce City level access scores are positively correlated with each other. Comparative analyses were performed both within and across urban settings to examine disparities in distributions of PHC services. It is found that neighbourhoods with poor accessibility scores in the main urban settings across Canada have further disadvantages in relation to population high health care needs.

**Conclusions:**

The results of this study show substantial variations in geographical accessibility to PHC services both within and among urban areas. This research enhances our understanding of spatial accessibility to health care services at the neighbourhood level. In particular, the results show that the low access neighbourhoods tend to be clustered in the neighbourhoods at the urban periphery and immediately surrounding the downtown area.

## Background

Access to primary health care (PHC) services is a pressing research and policy issue that is relatively unstudied in the context of urban settings, particularly with a focus on neighbouhoods and other small urban sub-units. One objective of the Canada Health Act (CHA or the Act) is “to protect, promote, and restore the physical and mental well-being of residents of Canada and to facilitate reasonable access to health services without financial or other barriers” [1]. It aims for a national health care system that is capable of providing universal, portable, comprehensive, publicly administrated, and accessible health services to all Canadians. The accessibility principle particularly “reasonable access to needed and appropriate health services” can be interpreted in many ways. The Canadian health care system, which is often held up as a model of a public, universal, and comprehensive system in international comparisons, went through an unprecedented series of changes in the 1990s as the federal government reduced its financial support to the provinces. Provincial governments have responded to these reduced levels of federal funding by undertaking aggressive restructuring tactics such as the closure of hospitals and the deinsuring of medical services from provincial health plans [2–4]. The result of this restructuring, as argued by the media, consumer groups, and indeed some health researchers, is an erosion of accessibility to health care services. Among the public, there is a growing perception that access to health care is declining [5]. In addition, research has shown that an increasing number of Canadians experience accessibility problems. For example, in 2001 approximately 12 percent of Canadians reported that they did not have a regular family doctor [6] while more recent data shows this figure had increased to 14.9 percent in 2014 [7]. Research also shows an increase in the number of Canadians reporting unmet health care needs. In 1994 approximately 5 percent of Canadians reported they did not receive health care when needed, in and by 2014 this figure had more than doubled [8–10].

While important for demonstrating increasing accessibility problems across the country, much of the research on access to health care services focused on national, sub-national (i.e., provincial in Canada, state in U.S.), and international levels [11, 12]. Yet, an increasing body of research in the UK [13–16], U.S., [17, 18], and Canada [19–21] points to the importance of neighbourhoods for shaping health and well-being. To date an abundance of research has focused on the links between neighbourhoods and health status (e.g., self-assessed, chronic conditions, cardiovascular disease) [22–24] as well as lifestyle behaviours (e.g., physical activity, diet) [25, 26]. In contrast, within the Canadian context, only a handful of studies have examined access to care in the context of neighbourhoods. For example, Roos and Mustard [27] found that in Winnipeg, Manitoba residents living in neighbourhoods in the bottom income quintile had increased levels of General Practitioner consultations, while residents living in upper income quintile neighbourhoods had higher rates of specialist utilization. However, in another study Chan and Austin [28] revealed that Ontario residents living in poor neighbourhoods had more referrals to specialists by primary care physicians. Research conducted by Yip et al., [29] shows that neighbourhood-level income is a strong predictor of physician utilization in Nova Scotia. Finally, in their study of access to care within four neighbourhoods in Hamilton, Ontario, Law et al., [19] found that both physician utilization and unmet health care needs vary by neighbourhood of residence, with a higher percentage of individuals living in the lowest income neighbourhoods reporting lower physician utilization and unmet health care needs. More research is needed to be focused on urban areas of varying sizes in order to understand the intra-urban patterns of geographic accessibility to health care.

Access to care is a complex concept and is interpreted differently by policy makers, researchers, and the general public [30, 31]. For example, access to health care is described as a relationship between characteristics of the service delivery system and of the population at risk to the actual utilization of services and consumer satisfaction [32]. Penchansky & Thomas [30], in describing access as the degree of “fit” between clients and the system, identify five key dimensions of access: availability, accessibility, accommodation, affordability, and acceptability. The first two dimensions (i.e., availability and accessibility) represent the geographic dimension of access. According to Penchansky and Thomas [30], availability describes the supply of health services in relation to the population in need, whereas accessibility describes the geographical location of health services in relation to the location of clients by considering the geographical factors (such as transportation, travel time, distance and cost). In the literature a distinction is made between potential and realized access. Potential access refers to the distribution or availability of health care services while realized access refers to the actual utilization of services [33–35]. Potential access is further divided in two components based on geographic (location and distance) and non-geographic barriers (such as socioeconomic status, income, age or gender) [33, 36]. This research uses the potential geographic access definition to examine the intra-urban distributions of PHC providers across Canada.

This study hypothesized that geographic accessibility to PHC services (accessibility scores) will vary considerably across the neighbourhoods in Canadian major urban settings and neighbourhoods with poor accessibility scores will have further disadvantages in relation to socially disadvantaged groups (presenting high health care needs). The specific objectives of this study are: 1) to examine the potential geographical accessibility to PHC services across Canadian urban areas to identify underservices or poorly served neighbourhoods; 2) to investigate the differences between the intra-urban patterns of spatial access score and simple physician-to-population ratio; and 3) to explore the association of poor accessibility scores with socially disadvantaged population groups.

## Methods

The research examines potential access to care across 14 Canadian urban areas, representing all 10 provinces; each city is a Census Metropolitan Areas (CMAs). Statistics Canada uses three main approaches to dividing Canada geographically for census purposes: urbanization level (e.g., CMA, Census Tract ‘CT’), legal boundaries (e.g., Census Subdivision ‘CSD’, health region), and electoral boundaries. The CMA is an urban area with at least 100, 000 populations and is “formed by one or more adjacent municipalities centred on a population centre (known as the core)” [37]. The number of various geographical units (CSD, city defined Neighbourhood and DA) are given in the Table 1. The included cities are Victoria and Vancouver, British Columbia; Calgary and Edmonton, Alberta; Saskatoon, Saskatchewan; Winnipeg, Manitoba; Hamilton and Toronto, Ontario; Ottawa–Gatineau, Ontario and Québec; Montréal and Québec City, Québec; Halifax, Nova Scotia; Saint John, New Brunswick; and St. John’s, Newfoundland. These urban areas have been selected for comparative purposes after considering their distinct characteristics. Among the study sites, Toronto with a population of 5.11 million has the highest population density (866.1 population per square kilometer) whereas Saint John’s (0.122 million population) has the lowest population density (36.4 population per square kilometer). Each urban area consists of at least one CSD (or Municipality, in more common terms) for which locally relevant neighbourhoods exist (for details, see Table 1). The number of neighbourhoods in each urban area are given in Table 1. In the study of local-level access to health care, the unit of analysis is critical, as the size and shape of the area chosen for investigation (e.g., county units, postal codes, census tracts, municipally-defined neighbourhoods) may produce different results. A recent study examining different aspects of access to health care at local scales, demonstrated that the size and shape of the selected neighbourhood matters [38]. There is a range of ways in which neighbourhoods are defined, including delineation using physical features, following administrative boundaries, using or aggregating census areal units, etc., and definitions that reflect residents’ perceptions about neighbourhoods. In recent years, the use of meaningful units of analysis in geographical research has been popularized [39–41]. In Canadian urban settings, locally developed (i.e., city/municipality defined) or relevant (or natural) neighbourhood units are argued to be more meaningful units of analysis for City planners (e.g., [42–45]), health workers, researchers than neighbourhoods defined by data availability (e.g., dissemination areas, census tracts, etc.) [39, 40]. In this research, city-defined neighbourhoods are used as unit of analysis.

**Table 1:** Information about the geographical units involved and family physicians by urban areas.

Urban Area (CMA)	*2011 population*	Areal Units			Family Physician	
		CSD	NH	DA	Practice	Physicians
		*n*	*n*	*n*	*sites*	*count*
Calgary, AB	1,096,184	1	223	1,588	466	1,070
Edmonton, AB	873,157	2	351	1,253	347	839
Halifax, NS	390,091	1	23	593	168	454
Hamilton, ON	656,574	2	208	1,083	264	552
Montreal, QC	1,886,481	16	118	3,201	635	1,542
Ottawa-Gatineau, ON & QC	1,148,740	2	93	1,769	448	1,196
Québec City, QC	672,136	4	50	1,151	217	751
Saint John, NB	82,010	2	33	156	60	87
Saskatoon, SK	221,849	1	82	360	68	234
St. John's, NL	180,396	5	145	300	62	196
Toronto, ON	2,615,060	1	325	3,685	1,435	2,579
Vancouver, BC	892,696	3	76	1,403	516	1,068
Victoria, BC	280,373	9	67	460	192	427
Winnipeg, MB	663,617	1	230	1,118	188	528
**Total**	**11,659,364**	**50**	**2,024**	**18,120**	**5,066**	**11,523**

In this study, we measured spatial accessibility to family physicians to examine intra-urban variations in access to care. In the Canadian context, primary care refers to first-point-of-contact health services between an individual and a health care practitioner such as a family physician, nurse practitioner, or pharmacist [46, 47]. The term family physician (or general practitioner) refers to a physician who has family medicine training (see, [48]). The location of physicians, particularly family physicians, plays a central role in health care delivery [34] as well as continuity of care [49, 50]. How physicians choose their practice sites can be influenced by environmental and or behavioral factors [34]. Environmental factors are related to area characteristics–current patterns of physician distribution, locations where businesses are allowed, potential patients, etc. [34, 51, 52]. “If health care and public health programs and services do not include a focus on the needs of disadvantaged individuals, populations, and communities, there is a risk of increasing rather than reducing health disparities” [53]. In this regard, the role of PHC providers is a crucial one [53, 54]. For this research, Information about physician’s practice sites were derived from individual profiles collected from the Provincial Colleges of Physicians and Surgeons. Only those physicians specified as Family Doctors/ Physicians, General Practitioners or Non-specialists and those who have their primary practice sites within the municipal boundaries of the study areas examined were considered. The Canadian Institute for Health Information (CIHI)’s report about Supply, Distribution and Migration of Canadian Physicians indicates that there were 40,781 family physicians or general practitioners (i.e., 50.1% of the physician workforce in Canada) in Canada and a majority of them were working in urban areas (i.e., 86.1% of family physicians in Canada) in 2014 [55]. In the 14 urban areas selected for this research, a total of 11,523 physicians providing PHC services located in 5,066 practice sites were included (see Table 1). The geographic coordinates for PHC practice sites were generated by applying an integrated geocoding process for maximum match rate (i.e., 100 percent) with reduced positional uncertainty [56]. Population data was taken from the 2011 Census of Canada. In Canada, Census data is disseminated at a wide range of geographic areas. Dissemination Area (DA), the smallest geographic area at which complete census data is released, was used in this study. The geographic coordinates for DAs are provided by Statistics Canada (as a part of the GeoSuite product).

In this study, spatial accessibility to family physicians was measured using a three-steps floating catchment areas (3SFCA) method [38, 40] to examine intra-urban variations in access to PHC services. The 3SFCA approach involves more complex calculations of access that use spatial interaction processes (e.g., distance decay) in the manipulation of supply and demand data at local scales [34–36, 57, 58]. The 3SFCA method is based on the floating catchment area (FCA) approach and is a modified version of an earlier model—Two Step Floating Catchment Area (2SFCA) method [36, 59–61]. Other methods using FCA approach are also available to measure geographic accessibility to healthcare services [62–67]. An in-depth review of the accessibility methods those are build on the FCA model can be found here [68, 69]. In short, there are two significant improvements to the original 2SFCA method that was introduced by Luo and Wang (36): 1) account for distance-decay within a catchment [57, 70] and 2) usage of variable catchment sizes [70, 71]. However, these modifications in the accessibility measures are crucial and meaningful in semi-urban, rural and remote areas whereas in case of densely populated areas such modifications are less functional. To-date, very few geographical measures have focused on examining spatial accessibility in an urban context [72]. Previous research has demonstrated that the 3SFCA method [38, 40, 72] is appropriate when measuring intra-urban geographic accessibility to health care because this better explains the geographic interaction between the health care access and demand. This method requires the application of Geographic Information Systems, an integrated computer-based set of tools for working with spatial data. Spatial data includes addresses, coordinates, and layers of thematic data (rivers, streets, landcover, etc.), each with accompanying non-spatial information. GIS supports data integration (combining address data with thematic data, like road networks and neighbourhood boundaries), analysis, and communication (often in the form of maps). Like other GIS-based methods for measuring geographic accessibility to health care, the 3SFCA method requires the location of health care services and population information associated with geographic areas [36, 38, 58, 60, 73].

In the first step, the 2SFCA places a buffer, or catchment around a point of health care supply, and calculates a provider-to-population ratio within it–a coverage approach [74]. In the second step, it then places a second buffer around a point of population demand, and sums the ratios from all provider points within that second buffer. The two-step buffering accommodates for health care being sought across areal unit borders (i.e., neighbourhoods) (For more details, see [75]). However, one limitation of the 2SFCA is its reliance on a single buffer size assuming access to be uniform within that buffer [63], which could be accommodated by deriving variable catchment size where target population or catchment area is already known [71]. This can be problematic when the units of analysis vary in size and can result in under and overestimation of access across units [76]. There are several cases in which this may occur. To avoid the methodological inaccuracies involved when examining variably sized neighbourhoods, we utilize the 3FSCA method described in detail by Bell, Wilson, Bissonette, & Shah [38] and Bissonnette, Wilson, Bell, & Shah [40]. In short, the first and second steps of the method are consistent with the 2SFCA analysis; however, as a point of population demand, we introduce a smaller census unit known as a Dissemination Area (DA), rather than using neighbourhood centroids. In an additional third step, an index of accessibility score (or access ratio) at the neighbourhood level is calculated by averaging the 2SFCA access ratios for all DAs falling within a neighbourhood. The third step results in a neighbourhood-level access ratio that is independent of neighbourhood size. This reduces methodological inaccuracies because the DAs used are smaller and more uniformly sized than neighbourhoods. In the case of this research employs a more moderate distance of 3km (in the first two steps of the method used; using road network to generate a catchment area) [50, 77, 78], based on the premise that local (i.e., neighbourhood) access to primary care is important if not universally put into practice during the family doctor selection process (see [79]). Research reported that utilization of alternative health care facilities over regular family physician in urban areas is observed when distance from place of residence to family physician practice is more than 3 km [50]. It is also noted that research conducted in urban areas generally involved moderate catchment distances (approx. 3km) [78]. However, it is important to acknowledge that these measures are limited to physical distance and cannot account for the amount of time it takes to travel set distances, a result of both physical and transportation barriers.

In the first stage of analysis, the 3SFCA method was applied to calculate potential geographic accessibility to family physicians in the 14 study areas. For this process, the following input datasets were used: a geocoded layer of PHC practice sites that represents PHC supply; the 2011 DA locations and associated population that represents demand for health care services [80]; and a digital neighbourhood (geographic) boundary file as a unit of analysis [81]. Catchment areas around all locations of PHC services and DA points required for the 3SFCA calculations were created using the service area function in Network Analyst, an extension of ArcGIS 10 software.

Next, Anselin’s local indicator of spatial association (LISA), a local form of Moran’s I, was applied for statistical confirmation and identification of clusters in urban fabric [82–84]. The LISA measures whether the 3SFCA accessibility score of a neighbourhood (i.e., index of spatial access to PHC services) is closer to the values of its neighbours or to the average of the urban area (see, [82]). In this study the univariate LISA tool provided in GeoDa software [83] was applied to each urban area separately and the following parameters were selected to compute global and local Moran’s I statistics: Queen’s case contiguity (1st order) to create a spatial weights matrix, a larger number of permutations (i.e., 999) to assess the sensitivity of results, and the significance filter set to .05. A spatial weights matrix is a way to numerically represent neighboring relationships and there are a number of ways to describe their proximity [85]. For example, contiguity or connectivity between neighbours, distance from centroid, or the exact number of neighbors are used in health geography. The contiguity can be further subdivided based on the nature of relationship such as Queen’s case contiguity that considers all possible connectivity between areas as a weight matrix which is normally suggested when dealing with the irregular boundaries [85]. GeoDa generated four different types of result graphs and maps: a significance map, a cluster map, a box plot and a Moran scatter plot along with a set of three output variables containing cluster related information for each unit of analysis [85].

In order to understand how certain population groups with high health care needs associate with the categories of accessibility scores, the following four socio-demographic variables were shortlisted based on theoretical significance and data availability [61, 86–89]: 1) population 15 years and over having no certificate, diploma or degree, 2) immigrants who came to Canada from 2001 to 2011, 3) lone-parents economic families, and 4) aboriginal identity population. Note that these variables were derived from 2011 National Household Survey (NHS). Census data was collected at the DA level [90] and aggregated to city defined neighbourhoods. It is noted that research highlights the limitations of using the 2011 voluntary NHS data such as data suppression of data due to low quality, data not available for 25 percent (of 4,567) CSDs [91–94].

## Results

**Fig 1:**
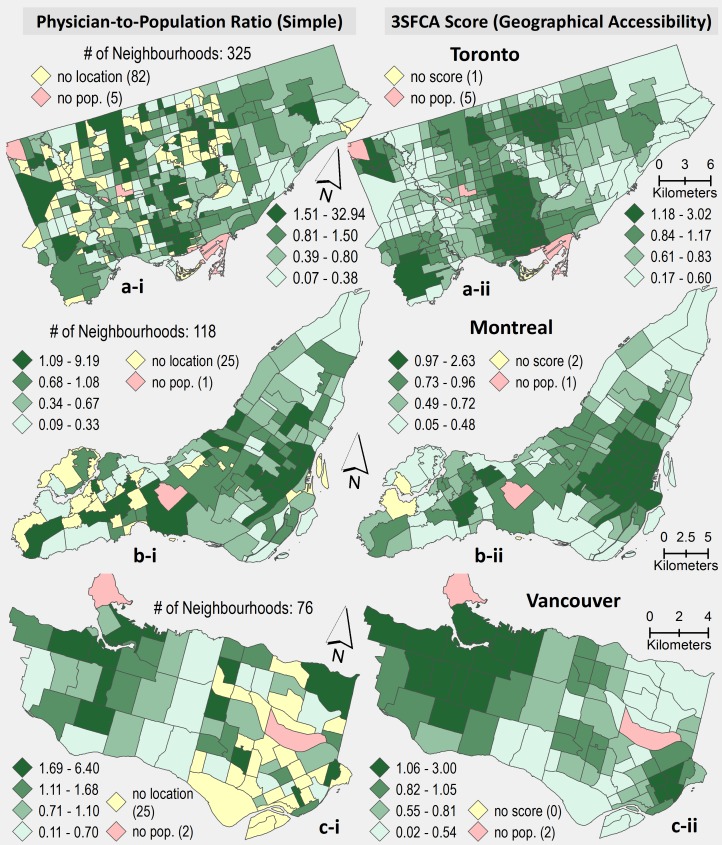
A comparison of population to physician ratios: simple ratio estimated at city level; City mean of simple ratio estimated at neighbourhood level; 3SFCA mean accessibility scores estimated at neighbourhood level.

The results of physician-to-population ratios for the 14 urban areas estimated using the 3SFCA method are presented in Table 1 (see also Fig 1). The column labeled Simple Ratio shows the simple calculations or physician to population ratios at the city scale (i.e., [PHC physicians in a City / 2011 Census population of that City] *1000) while the column labeled Mean Neighbourhood Simple Ratio is initially measured at the neighbourhood level using the same formula. The column labeled Mean 3SFCA Score shows the 3SFCA calculations–the index of spatial access to PHC services. The basic difference between these ratios are that the Mean 3SFCA Score and Mean Neighbourhood Simple Ratio were calculated at the neighbourhood level and then averaged out by urban areas, while the Simple Ratio was calculated only at the City scale using the total number of physicians and the total population in the respective urban area. It is important to demonstrate the difference between these methods first.

**Fig 2:**
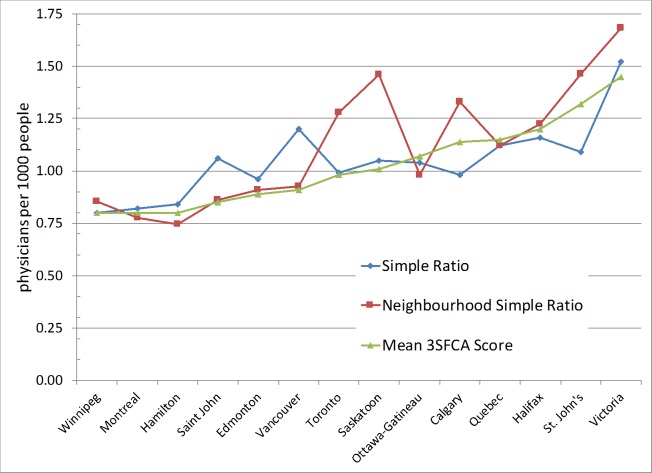
A comparison of physician-to-population ratio between simple ratio calculated using ([Physicians in an area/population of the area] * 1000) (in left column), and b) three- step floating catchment areas (3SFCA) method at the neighbourhood level (in right column) in three Canadian cities (a. Toronto, b. Montreal, and c. Vancouver).

As discussed in the Methods section above, a simple ratio method is suitable for those studies considering large regions as the unit of analysis including provinces, Census divisions, census subdivisions/municipalities, and census metropolitan areas. There are two issues associated with the simple ratio method (i.e., no values are assigned to a large number of units of analysis, and some unit of analysis have very high values) when one applies this process at a local scale to neighbourhoods, census tracts, wards, etc. (see Table 2). To illustrate this, both methods (i.e., simple ratio, and 3SFCA) are applied to the following cities: Toronto, Montreal, and Vancouver (see Fig 2) where a large number of analytical units are without physician-to-population ratios (i.e., 82, 25, 25 neighbourhoods in Toronto, Montreal, and Vancouver respectively). This is a typical problem in using the simple ratio method with small local units. Secondly, it provides very high ratio values (for example, 32.9, 9.2, 6.4 physicians per 1000 population in the case of Toronto, Montreal, and Vancouver respectively) and in total, there are eight urban areas with ratio values of more than 10 physicians per 1000 people. In contrast, the 3FSCA method accounts for the geographic reality of people moving around their local area to access services by calculating access based on nearby neighbourhoods and PHC. Visually, this has the effect of geographically smoothing rates of access. The three methods, Simple Ratio (A), Neighbourhood Simple Ratio (B), and 3SFCA (C), produce City level access scores that are positively correlated with each other (Pearson correlation coefficient, r_AB_ = 0.661, P = 0.01; r_AC_ = 0.785, P = 0.001; r_BC_ = 0.843, P <0.001).

**Table 2:** Summary of physician-to-populations ratios by urban areas.

Urban Area	2011 Census subdivisions		Family physicians		Physicians per 1000 Population				
			Practice Locations	Physicians	Simple Ratio^	Mean NH Simple Ratio^^		3SFCA Score^^^	
	*n*	*population*	*n*	*n*	*n*	*mean*	*max*.	*mean*	*max*.
Calgary, AB	1	1,096,184	466	1,070	0.98	1.33	34.23	1.14	4.54
Edmonton, AB	2	873,157	347	839	0.96	1.18*	57.42*	0.89	2.95
Halifax, NS	1	390,091	168	454	1.16	1.22	7.24	1.20	4.20
Hamilton, ON	2	656,574	264	552	0.84	0.74	10.22	0.80	2.88
Montreal, QC	16	1,886,481	635	1,542	0.82	0.78	9.19	0.80	2.63
Ottawa-Gatineau, ON & QC	2	1,148,740	448	1,196	1.04	0.98	6.12	1.07	2.99
Québec City, QC	4	672,136	217	751	1.12	1.12	5.47	1.15	3.32
Saint John, NB	2	82,010	60	87	1.06	0.86	5.59	0.85	3.01
Saskatoon, SK	1	221,849	68	234	1.05	1.46	14.76	1.01	2.92
St. John's, NL	5	180,396	62	196	1.09	1.46	40.65	1.32	3.85
Toronto, ON	1	2,615,060	1,435	2,579	0.99	1.28	32.94	0.98	3.02
Vancouver, BC	3	892,696	516	1,068	1.20	0.93	6.40	0.91	3.00
Victoria, BC	9	280,373	192	427	1.52	1.68	12.71	1.45	3.50
Winnipeg, MB	1	663,617	188	528	0.80	0.85	19.15	0.80	3.03
**Total**	**50**	**11,659,364**	**5,066**	**11,523**	**0.99**	**1.14**		**0.99**	

^Estimated using the following Simple ratio formula: PHC physicians in a city / 2011 Census population of the city] X 1000

*^^*Estimated using the same simple ratio at neighbourhood (NH) level data

^^^ Estimated using the 3SFCA method

* a very high value ‘400’ is not included.

To analyze the distribution of geographical accessibility to PHC services between the urban areas included in the study, we used the Kruskal-Wallis one-way analysis of variance by ranks to test whether there are variations between the urban areas on 3SFCA accessibility scores. The results show variations across the 14 urban areas (H = 77.865, 13 d.f., p < 0.001). Variations in geographical accessibility to PHC services between urban areas within the same province are evident (i.e., Victoria and Vancouver, British Columbia; and Toronto and Hamilton, Ontario).

**Fig 3:**
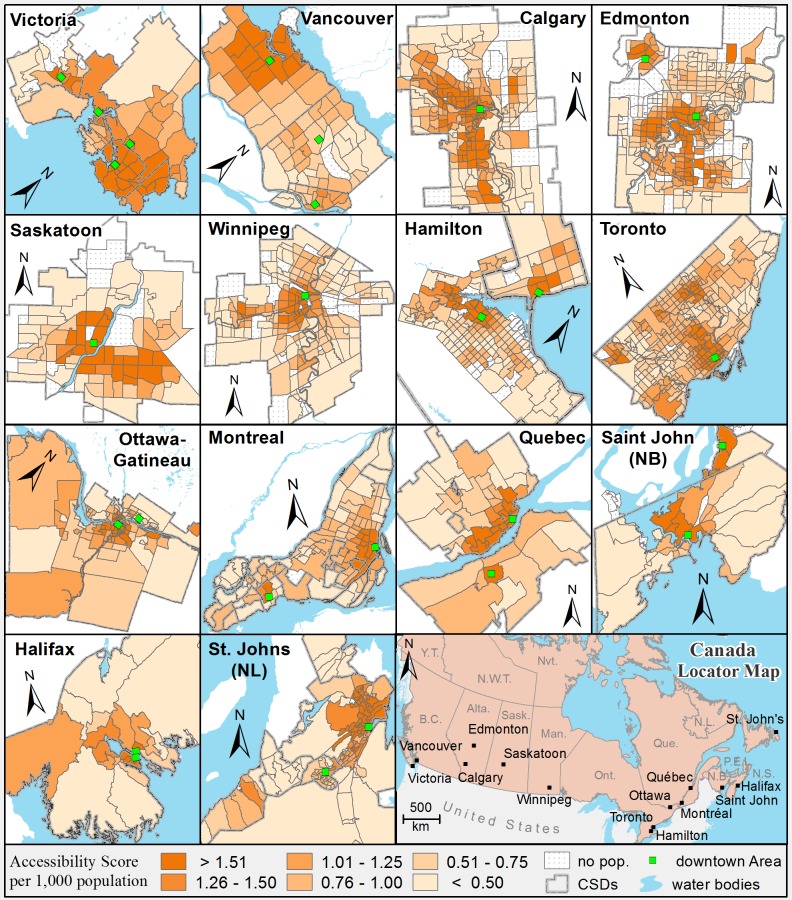
Spatial accessibility to PHC services by urban areas at the neighbourhood level estimated using the three steps floating catchment areas method (2011 DA Population, 3km road network buffer distance).

The geographic distributions of 3SFCA accessibility scores in the 14 urban areas are shown in Fig 3. The accessibility scores are categorized into six manually defined classes: less than 0.50; 0.50–0.75; 0.76–1.00; 1.01–1.25; 1.26–1.50; 1.51 and above. This is done to emphasize a particular range of values, above or below a threshold value such as one health care provider-per-1000 people—a measure that may be more meaningful to policy makers, health planner, and local residents. The first two classes, labeled as < 0.50 and 0.50–0.75, represent the neighbourhoods with the lowest accessibility to PHC services. Population proportions (in percentages) that fall into these six categories are given in Table 3. For all measures, higher numbers represent better access to PHC services.

**Table 3:** Proportion (%) of population in urban areas by group of accessibility score and LISA clusters.

Access Score (3SFCA)	Cluster	Cal	Edm	Hal	Ham	Mon	Ott	Que	S John	Sas	St. John's	Tor	Van	Vic	Win	Total
		%	%	%	%	%	%	%	%	%	%	%	%	%	%	%
**<0.5**	LL	46.9	28.6	45.2	22.3	36.4	16.9	38.7	10.1	37.4	79.6	75.6	65.5	61.8	22.5	40.4
	LH	0.0	0.6	0.0	0.0	0.1	0.0	0.0	0.0	0.0	0.0	0.0	0.0	0.0	0.0	0.1
	NS	53.1	70.9	54.8	77.7	63.4	83.1	61.3	89.9	62.6	20.4	24.4	34.5	38.2	77.5	59.5
<0.5		38.7	29.9	29.9	31.4	29.5	16.4	19.0	31.4	29.2	32.6	12.6	7.4	6.4	38.3	**23.1**
**0.5–0.75**	HH	0.0	0.0	0.0	0.7	0.0	0.0	0.0	0.0	0.0	0.0	0.0	0.0	0.0	0.0	0.0
	LL	14.0	11.8	0.0	0.4	8.8	22.8	25.7	0.0	0.0	48.8	43.0	9.5	0.0	2.5	20.4
	LH	0.0	0.0	0.0	5.7	0.0	0.0	0.0	0.0	0.0	0.0	0.0	0.0	0.0	0.0	0.3
	NS	86.0	88.2	100	93.2	91.2	77.2	74.3	100	100	51.2	57.0	90.5	100	97.5	79.3
0.5–0.75		16.1	18.2	14.4	19.8	22.4	22.1	26.3	6.3	23.0	9.4	27.7	24.8	11.2	24.8	**22.2**
**0.75–1.0**	HH	0.0	5.3	0.0	3.8	0.0	0.0	0.0	0.0	0.0	0.0	0.0	8.6	0.0	0.0	1.3
	LL	2.1	0.0	0.0	0.0	0.0	3.5	21.0	0.0	0.0	2.6	2.8	0.0	11.9	0.0	2.7
	LH	2.9	0.0	0.0	0.0	0.0	0.0	0.0	0.0	3.8	0.0	0.0	0.0	0.0	0.0	0.2
	HL	0.0	0.0	0.0	0.0	13.8	0.0	0.0	0.0	0.0	0.0	0.0	0.0	0.0	5.9	3.2
	NS	95.0	94.7	100	96.2	86.2	96.5	79.0	100	96.2	97.4	97.2	91.4	88.1	94.1	92.7
0.75–1.0		9.9	10.6	8.5	21.0	27.3	24.2	19.8	17.2	9.2	14.5	26.4	25.9	11.7	11.1	**20.5**
**1.0–1.25**	HH	0.0	27.8	0.0	18.0	29.2	6.9	0.0	31.3	0.0	0.0	1.5	28.4	0.0	7.0	11.9
	LL	0.0	0.0	0.0	0.0	0.0	0.0	0.0	0.0	0.0	0.0	0.0	0.0	4.9	0.0	0.2
	LH	0.0	0.0	0.0	0.0	0.0	0.0	0.0	0.0	0.0	3.6	0.0	0.0	0.0	0.0	0.0
	HL	1.2	0.0	0.0	1.5	0.0	0.0	0.0	0.0	0.0	0.0	0.0	0.0	0.0	0.0	0.1
	NS	98.8	72.2	100	80.5	70.8	93.1	100	68.7	100	96.4	98.5	71.6	95.1	93.0	87.8
1.0–1.25		4.2	13.9	17.9	7.5	8.0	10.6	2.3	3.9	5.8	8.1	12.4	10.8	13.7	6.0	**9.5**
**1.25–1.5**	HH	7.3	47.5	0.0	31.5	85.3	30.4	0.0	-	100	13.1	28.6	78.1	0.0	58.9	27.7
	HL	0.0	0.0	0.0	0.0	0.0	13.7	0.0	-	0.0	0.0	0.0	0.0	0.0	0.0	1.7
	NS	92.7	52.5	100	68.5	14.7	55.9	100	-	0.0	86.9	71.4	21.9	100	41.1	70.5
1.25–1.5		10.3	7.8	9.3	3.7	1.5	7.7	6.5	0.0	3.4	10.2	6.0	1.9	17.6	6.3	**5.9**
**>1.5**	HH	63.0	84.8	77.7	75.6	89.3	79.7	65.0	9.2	42.9	52.8	90.9	100	73.9	73.6	79.0
	HL	0.0	0.8	0.0	0.0	0.0	0.0	0.0	0.0	0.0	0.0	0.0	0.0	1.5	0.0	0.1
	NS	37.0	14.4	22.3	24.4	10.7	20.3	35.0	90.8	57.1	47.2	9.1	0.0	24.5	26.4	20.9
>1.5		20.8	19.7	19.9	16.6	11.4	19.0	26.1	41.2	29.3	25.2	14.9	29.3	39.4	13.5	**18.8**

Note: LISA = local indicators of spatial autocorrelation; LISA clusters are depicting the neighbourhoods of significant local Moran’s I statistics–spatial association: spatial clusters [LL (Low surrounded by low values) and HH (High surrounded by high values)] and spatial outliers [LH (Low surrounded by high values), HL (High surrounded by low values)], and NS (Not significant); Cal = Calgary, Edm = Edmonton, Hal = Halifax, Ham = Hamilton, Mon = Montreal, Ott = Ottawa-Gatineau, Que = Quebec, S John = Saint John, Sas = Saskatoon, Tor = Toronto, Van = Vancouver, Vic = Victoria, Win = Winnipeg.

**Fig 4:**
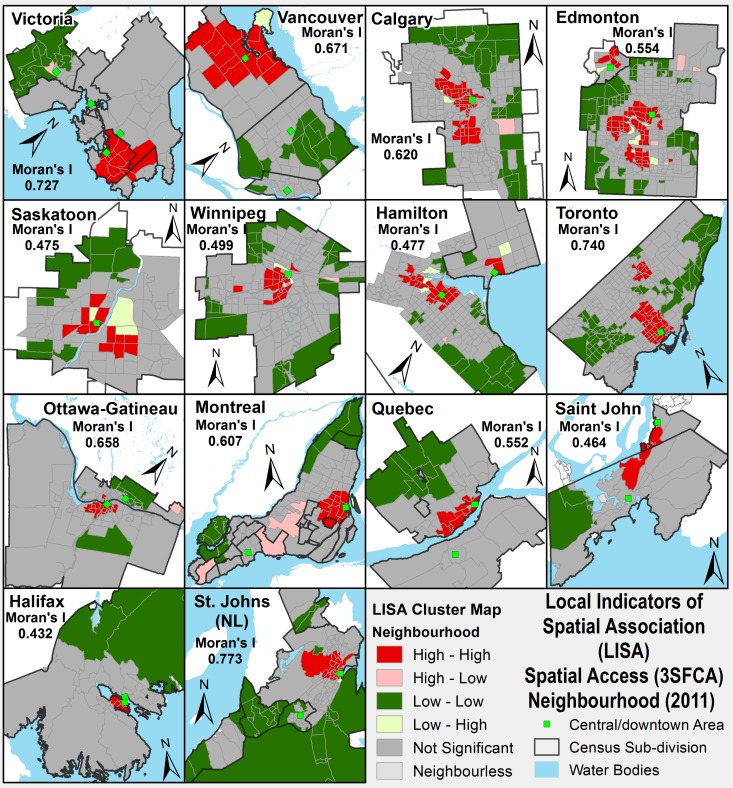
LISA cluster map of spatial accessibility to PHC services (physicians to population ratio) by urban areas. Global Moran’s I of urban areas as given are found statistically significant (pseudo-significant = 0.05).

The LISA cluster maps for all 14 urban areas with Global Moran’s I results, providing initial evidence of clustering of accessibility to PHC services, are shown in Fig 4. The Moran’s I value vary from a minimum of 0.432 for Halifax to a maximum value of 0.773 for St. John's. Spatial clusters based on positive spatial association are labeled as ‘High-high’ and ‘Low-low’ referring to neighbourhoods that have high (or low) spatial accessibility scores and are surrounded by high (or low) accessibility values. Whereas spatial clusters (also called spatial outliers) based on negative spatial association are indicated as ‘High-low’ and ‘Low-high’ refer to neighbourhoods that have high (or low) accessibility that are surrounded by low (or high) accessibility values. As the results of the spatial autocorrelation (Moran’s I–a statistical diagnostic) are interpreted within the context of its null hypothesis, a not significant label are used to referred to neighbourhoods whose p-value is not statistically significant—cannot reject the null hypothesis. In other words, the spatial distribution of neighbourhood values is the result of random spatial processes [84, 85].

The percentages of census based socio-demographic variables within broad categories of accessibility scores (i.e., <0.75; 0.75–1.25; >1.25) are presented in Table 4 for comparative purposes. The following two types of percentages are used in Table 4: percentage out of total population (R), and percentage out of total population of respective socio-demographic factor. For example, in case of Calgary, 60.6 percent (P) of total recent immigrants in Calgary that are 12.7 percent (R) of the respective total population are found within <0.75 category of neighbourhood access scores. It is found that high percentages of these population groups those comparatively with high health care needs fall in neighbourhoods with poor (<0.75) accessibility scores (e.g., Population by Aboriginal Identity–about 60.3% of total aboriginal population of Calgary city fall within poor access category (<0.75) which is 2.4% of the total population of the respective category).

**Table 4:** Percentages of census-based socio-demographic variables within broad categories of accessibility scores by urban areas (where R represents the % out of total population and P represents % out of total population of respective socio-demographic factor).

Access Score (3SFCA)		Cal	Edm	Hal	Ham	Mon	Ott	Que	S John	Sas	St. John's	Tor	Van	Vic	Win	Total
a. Population 15 years and over having no certificate, diploma or degree																
<0.75	R	16.7	19.3	19.5	19.4	22.5	15.3	14.8	19.9	17.9	16.6	20.6	16.5	13.5	19.1	**19.0**
	P	59.6	53.2	53.5	50.9	61.2	38.9	42.8	35.9	53.5	41.4	47.3	38.3	18.8	60.9	**50.5**
0.75–1.25	R	14.0	17.1	15.8	21.2	16.6	14.0	17.2	25.9	15.3	16.7	17.6	16.5	13.8	21.9	**16.9**
	P	13.3	24.5	26.3	30.9	31.3	33.5	24.8	25.6	13.7	23.5	38.9	44.0	28.0	19.0	**30.2**
>1.25	R	12.4	13.5	10.6	17.3	10.5	14.7	14.7	19.9	16.3	15.8	11.0	7.6	11.6	20.0	**12.7**
	P	27.1	22.4	20.2	18.3	7.5	27.6	32.5	38.5	32.8	35.1	13.8	17.8	53.3	20.1	**19.4**
b. Recent Immigrants (2001–2011)																
<0.75	R	12.7	9.3	1.2	4.1	12.3	5.5	1.0	0.4	6.4	0.4	15.5	15.6	1.4	8.8	**10.0**
	P	60.6	48.5	20.3	42.7	49.0	35.7	22.2	12.2	53.7	22.6	39.8	36.8	8.1	60.3	**44.6**
0.75–1.25	R	9.7	8.8	3.4	4.7	14.4	5.9	0.8	1.0	5.1	0.4	16.7	13.7	4.0	8.3	**11.3**
	P	11.8	23.3	34.6	27.4	38.8	34.7	8.2	17.3	12.4	10.9	41.2	37.0	32.2	15.3	**33.0**
>1.25	R	10.4	9.7	4.1	7.5	12.5	6.7	4.5	2.1	6.5	1.4	14.4	11.6	3.3	11.7	**9.5**
	P	27.6	28.2	45.2	29.9	12.2	29.7	69.7	70.4	33.9	66.5	19.0	26.2	59.7	24.4	**22.4**
c. Lone-parent economic families																
<0.75	R	11.0	13.3	11.8	12.3	17.9	12.9	10.9	12.6	12.3	10.1	18.2	13.1	7.6	12.6	**14.3**
	P	60.5	50.9	47.0	49.3	57.8	39.1	44.7	30.6	58.6	37.2	46.5	37.8	12.7	58.0	**48.7**
0.75–1.25	R	9.4	13.4	13.7	14.2	16.4	12.8	12.4	19.6	9.5	11.5	15.9	12.1	11.9	18.2	**14.4**
	P	13.3	25.9	30.9	30.8	33.8	34.6	23.8	24.7	12.9	22.1	38.7	39.5	27.4	21.5	**31.3**
>1.25	R	9.0	11.8	10.2	14.4	12.9	14.3	13.4	19.4	10.7	14.8	12.4	9.1	13.0	17.0	**12.3**
	P	26.2	23.2	22.0	19.9	8.4	26.2	31.5	44.7	28.5	40.7	14.8	22.8	60.0	20.5	**20.1**
d. Population by Aboriginal Identity																
<0.75	R	2.4	4.8	2.0	1.0	0.2	2.0	0.4	1.3	9.7	1.6	0.4	1.1	3.5	9.1	**2.1**
	P	63.6	49.6	51.4	47.3	52.3	43.3	60.0	37.3	54.3	47.7	53.4	26.6	20.5	53.1	**49.9**
0.75–1.25	R	1.9	4.6	1.8	1.2	0.1	1.6	0.3	1.1	5.9	1.6	0.2	1.5	2.2	13.4	**1.5**
	P	13.2	24.2	26.7	32.0	34.6	30.2	19.9	17.1	9.6	25.8	27.5	43.6	18.2	21.0	**22.6**
>1.25	R	1.5	4.6	1.3	1.2	0.2	1.8	0.2	1.5	10.4	1.0	0.3	1.3	3.3	14.5	**2.2**
	P	23.2	26.2	21.9	20.7	13.0	26.5	20.1	45.6	36.1	26.4	19.1	29.8	61.3	25.9	**27.5**

Note: Cal = Calgary, Edm = Edmonton, Hal = Halifax, Ham = Hamilton, Mon = Montreal, Ott = Ottawa-Gatineau, Que = Quebec, S John = Saint John, Sas = Saskatoon, Tor = Toronto, Van = Vancouver, Vic = Victoria, Win = Winnipeg.

## Discussion

The main goal of this research was to measure the geographical accessibility to family physicians by applying the 3SFCA method and to identify neighbourhoods having poor accessibility to PHC services and their spatial patterns in urban settings. This research compares the results in 14 Canadian urban areas at both the urban area and neighbourhood level. Although the study has successfully demonstrated that considerable spatial variations in potential geographical accessibility to PHC services exist within and across urban areas, it has certain limitations in terms of physician data selection and processing.

It should be noted that only those physicians who fall in the category of *Family Doctors*, *Family Physicians*, *General Practitioners*, or *Non-Specialists* and have valid geocodeable addresses are included in this study. Physicians having no address (68 in total) and having Post Office Box (P.O. Box) information (202 physicians) were removed. This omission of non geocodeable addresses may underestimate the accessibility to PHC services. The presence of such addresses in the analysis would increase the positional uncertainty of geocoded locations [95] which could change the overall research findings [96]. It should also be noted the DA centroids, which represent the health care demand sites and geocoded locations of PHC services that may carry some positional errors, were used in the 3SFCA method and may generate some biases in the research findings (such as, considerable impact on the results of spatial regression analysis [97], inaccurate results at finer-scale analysis [98], etc. are reported; for a detailed overview of the potential biases in health research, see [99]). As this study did not consider population and physician data for neighbouring municipalities in all urban areas, edge effects may also be present. Geographical accessibility to only those physicians who accept new patients could be calculated to demonstrate the shortage of PHC services in urban areas, however in this research we are more interested in demonstrating the benefit of the 3SFCA accessibility score in identifying under serviced or poorly-served neighbourhoods, exploring the spatial patterns within urban settings, and comparing the results at both city and local levels as well.

With respect to the spatial distribution of access within the urban areas, the results show that the highest access neighbourhoods tend to be clustered in the central or downtown areas of all cities with accessibility levels decreasing in the neighbourhoods immediately surrounding the downtown area, and further decreasing at the urban periphery (see Fig 3). However, a slight variation in spatial distribution of access is evident in some Census Metropolitan Areas (CMAs) where multiple downtown or core areas are present (for example; Hamilton, Vancouver, Edmonton, etc.). Overall, 23.1 and 22.2 percent of the total population (i.e. 2,697,493 and 2,589,539 out of 11,659,364) fall into the first (< 0.50) and second (0.50–0.75) categories respectively, with lower access to PHC. The largest population proportions (63.1 percent of 663,617 population and 54.8 percent of 1,096,184 population) in categories less than 0.50 and 0.50–0.75 are found in Winnipeg and Calgary respectively (see Table 3).

The outcomes of cluster analysis show that the neighbourhoods with high 3SFCA values are located in the downtown core areas of urban areas and underserviced neighbourhoods (i.e., low 3SFCA values) in most urban areas are located along borders of the municipalities or the most outlying sections of the city. Table 3 shows the results of spatial clusters of the 3SFCA accessibility score and the population proportion (in percentage) for each category of accessibility scores (there are in total six categories: less than 0.50; 0.50–0.75; 0.76–1.00; 1.01–1.25; 1.26–1.50; 1.51 and above, as given in Fig 3) are further divided by LISA clusters. For example, 23.1 percent of the total population in all urban areas that fall in the lowest accessibility category (i.e., accessibility score less than 0.50) is further divided into three groups: 40.4 percent of this 23.1 percent population fall in the Low-low cluster, 0.1 percent in the low-high and 59.5 percent in non-cluster neighbourhoods as shown in Table 3. Overall, 40.4, 20.4, and 2.7 percent out of the total percent of the first three categories (first (< 0.50), second (0.50–0.75), and third (0.75–1) respectively) fall in the low-low cluster type. A possible explanation for these results that show higher access to family physicians in the core neighbourhoods might be related to the environmental (availability of commercial / business spaces across urban areas) and or behavioral factors [34, 51, 52]. Urban core neighbourhoods (i.e., central business districts “CBDs” or city centre) have more space for businesses while outlying neighbourhoods / areas split their space between residential and non-residential (such as urban services zones, commercial zones, open spaces) areas [100] and are more extensive so business / urban services might tend to be more spread out. The behavioral factors are more related to personal decision regarding choice of practice location. Joseph & Phillips (1984) focused on the individual preferences in the context of locational choice of physicians identifying three important components of attitude formation: personal, professional, and class or lifestyle [34]. In a survey of practicing physicians in the province of British Columbia, Kazanjian & Pagliccia, (1996) found that physicians, regardless of urban and rural location, ranked spousal influence to be the most important in the choice (decision) of the practice location [51].

Neighbourhoods with poor accessibility scores are found in major urban settings across Canada that have further disadvantages in relation to high health care needs (i.e., socially disadvantaged groups). Overall, 50.5 percent of total population having no high school education (i.e., 19.0% of the total population in that category), 44.6 percent of recent immigrants from 2001 to 2011 (i.e., 10.0% of the total population in that category), 48.7 percent of lone-parents (i.e., 14.3% of the population in that category), and 49.9 percent of aboriginal identity population (i.e., 2.1% of the total population in that category) are found in neighbourhoods with poor accessibility (<0.75 range). In Calgary, Saskatoon, and Winnipeg, over 50 percent of the total population in all four socio-demographic groups also live in neighbourhoods with poor accessibility scores (<0.75) and in Edmonton, Hamilton, and Montréal, almost 50 percent of these same population groups living in neighbourhoods with poor accessibility scores. It is found that Ottawa–Gatineau, Saint John, St. John’s, Victoria, and Vancouver, where less than 50 percent of the total population in all four groups with few exception (Halifax, Québec City, and Toronto) fall in neighbourhoods with poor accessibility scores (<0.75). Since there is higher access to family physicians in the core neighbourhoods then outlying neighbourhoods, the findings that suggest socially disadvantaged groups (population having no high school education and lone-parents) have lower values in most of the CBDs seem to be consistent what Broadway [101] indicated the existence of two types of CBD (or inner city) based on deprivation levels among Canadian cities. In case of neighourhoods with high concentration of recent immigrants (or visible minorities) those normally prefer to settle in new or outlying neighbourhoods with affordable housing [102] are associated with poor access to family physicians. In general, therefore, the findings of this study have a number of policy implications for improving geographic accessibility to health care services in Canadian urban settings. Consistent with the aims of Canada’s Strategy for Patient-Oriented Research (SPOR) [103, 104] and for better access to PHC services in order to decrease emergency visit to seek PHC services that could be provided by family physicians [105–107], this study provides evidence to inform progressive and accessible PHC, all fundamental to actualizing the Act and benefiting the health of urban residents. Such as neighbourhoods with poor geographical accessibility to PHC services and high health care needs can be focused in the process of urban area development by city planners by ensuring/ providing / allocating business spaces for family physicians.

This research demonstrates the benefit of using the 3SFCA method over simpler approaches in urban areas by providing similar results of city-level physician-to-population ratios with the advantage of intra-urban measurements. However, some variations are observed at City level (i.e., urban areas) physician-to-population ratios where the 3SFCA method over (such as St. John’s and Calgary) and under (such as Vancouver and Saint John), estimates access to PHC services. The results do show variations across the 14 urban areas (see Fig 2). For example, Winnipeg appears to have the lowest levels of access to PHC physicians of all 14 urban areas while Victoria has the highest in both methods (Simple Ratio and 3SFCA); the differences are 0.74 and 0.65 respectively.

## Conclusion

This paper sought to address the following key issues related to geographic accessibility to primary health care (PHC) services across Canadian urban settings: first, to measure the geographic accessibility to and of family physicians (i.e., accessibility score) using a GIS based three-step floating catchment areas (3SFCA) method; and second, based on accessibility score calculated, identify under-served (or poorly served) neighbourhoods (or population) in the study areas. This study found considerable intra-urban variations in potential geographical accessibility to family physicians across Canadian urban areas and highlights neighbourhoods within urban fabric where there is disparity in geographical accessibility to PHC services. The findings from this study contribute to the health geography literature in several ways. The present study confirms previous findings [38, 40, 108] and contributes additional evidence that the 3SFCA method is an important addition to the health geography particularly in health services research where intra-urban disparity in geographical accessibility to health care services may help to examine more closely the underserved population and interventions to improve the health of urban disadvantaged population [109–111]. Needs to link with the comparison of different accessibility measures and reasons why 3sfca methods is most appropriate for urban analysis. In addition, the 3SFCA method has great potential to be used in other areas such as measuring spatial accessibility to dental, HIV and rehabilitation, and mental health care services.

Information on geographic accessibility to health care services should be measured on a regular basis to observe changes in under-serviced regions and shared with physicians; particularly those who are looking to start new practice, those who are in training/newly graduated, or those who wish to change their practice locations. This information on the distribution of health care services and their proximity to homes would be useful for policymakers, researchers, city planners, community workers, and those residents who need services. In this regard, a standardized and compatible physician and clinic database (or directory) at a national level that is well linked with provincial databases (College of Physicians and Surgeons) would be helpful in measuring accessibility at local scales and would aid in mapping service locations to reduce health inequalities. Further, information on a physician’s working hours, hours by location, language skills, whether they are accepting patients etc., as a part of this national physician database would be beneficial in exploring other aspects of geographic accessibility and its links with contextual and socio-demographic factors as well. Another important practical implication is that intra-urban patterns of geographical accessibility to PHC services can be utilized in physician workforce planning by provincial and regional decision makers, and in the process of urban area development by city planners. Future research could investigate the relationship between geographic accessibility to PHC services and socio-demographic characteristics in urban settings.

## Abbreviations

CSD: Census subdivision
CMA: Census Metropolitan Area
CIHI: Canadian Institute for Health Information
DA: Dissemination Area
LISA: Local indicator of spatial association
NHS: National Household Survey
PHC: Primary health care
3SFCA: Three-Step Floating Catchment Area
